# Investigation of the SPAG5 gene expression and amplification related to the NuMA mRNA levels in breast ductal carcinoma

**DOI:** 10.1186/s12957-020-02001-8

**Published:** 2020-08-24

**Authors:** Zeinab Mohamadalizadeh-Hanjani, Shirin Shahbazi, Loabat Geranpayeh

**Affiliations:** 1 Department of Medical Genetics, Faculty of Medical Sciences, Tarbiat Modares University, Tehran, Iran; 2 Department of Surgery, Sina Hospital, Tehran University of Medical Sciences, Tehran, Iran

**Keywords:** SPAG5, NuMA, Gene expression, Gene amplification, Breast ductal carcinoma

## Abstract

**Background:**

The cell proliferative markers are very important in breast cancer. Since SPAG5 and NuMA proteins play a significant role in the mitosis regulatory network and cell division, we aimed to study their mRNA levels as well as SPAG5 gene amplification correlated to clinicopathological status in ductal carcinoma of the breast.

**Methods:**

SPAG5 and NuMA gene expressions were investigated in 40 breast cancer tissues and normal adjacent tissues via real-time PCR. PUM1 was selected as the reference gene. QMF PCR method was applied to study SPAG5 gene amplification and AGBL2, BOD1L, and POR were designated as internal control genes. Gene amplification was determined by calculating a dosage quotient for each DNA fragment.

**Results:**

Increased SPAG5 mRNA expression was detected in breast cancer tissues (*p* = 0.005) and related to tumor size. No significant difference was observed between NuMA gene expression level in tumor tissue and the normal adjacent tissue (*p* = 0.56). However, we observed that NuMA expression was significantly increased in ER-positive tumor tissues. There was no clear correlation pattern between SPAG5 and NuMA mRNA levels (*r* = 0.33). Seventeen percent of tissues showed complete amplification in SPAG5 gene fragments.

**Conclusion:**

Our results were consistent with the previous publications regarding SPAG5 gene expression and amplification in breast cancer with an emphasis on the prominent role of this protein in tumor pathogenesis. Our results failed to yield any correlation between SPAG5 and NuMA mRNA levels which implies independence of these genes in breast cancer pathogenesis.

## Introduction

Breast cancer is the most common cancer type and the second leading cause of cancer death in women worldwide. Numerous studies have been conducted to find the cellular and molecular biomarkers to improve early detection and management of the disease and reduce its mortality rate. The study published by Abdel-Fatah et al. has drawn attention to sperm-associated antigen 5 (SPAG5) as a new prognostic biomarker in breast cancer. Using the interactome map, they showed a prominent role for SPAG5 in patient’s response to chemotherapy and survival [1].

SPAG5 gene is located on chromosome 17(17q11.2) comprising 24 exons with nine reported mRNA splice variants. It is categorized among conserved genes as it has been present since the common ancestor of chordates. The 1193 amino-acids protein of SPAG5 is a homodimer which binds to microtubules in the mitotic spindle and regulates its dynamic function [2]. SPAG5 is one of the essential components required for chromosome alignment, normal timing of sister chromatid segregation, and spindle pole formation. SPAG5 protein is expressed in a cell cycle-dependent manner with the highest level at telophase [3].

In non-mitotic cells, various stimuli upregulate SPAG5 expression. SPAG5 protein plays multiple roles in different pathways of cell growth and differentiation including PI3K/Akt. Upon cell oxidative stress, SPAG5 prevents apoptosis via mTORC1 suppression. Significant evidence confirms that the PI3K/AKT/mTOR pathway is the most common defected signaling cascade in breast cancer [4]. Increased SPAG5 mRNA expression in tumor tissues was reported in different cancers such as cervix, prostate, liver, lung, and bladder [5–7], indicating the higher SPAG5 levels the poorer prognosis and patient survival [1, 8]. Despite several reports of increased SPAG5 expression in cancer, the cellular and molecular events affected by this increase are still unclear. It is not well elucidated whether spindl-associated proteins are involved as much as cell signaling cascades.

One of the important SPAG5 interactors in cytokinesis is the nuclear mitotic apparatus (NuMA) protein which directly binds and recruits SPAG5 to the mitotic spindle. This interaction is reciprocal in a way that the lack of SPAG5 interferes with the appropriate NuMA spindle distribution and function [9]. NuMA gene is located on 11q13 with 38 exons that encodes a large 2115 amino-acids protein. It acts as a microtubule-binding protein involved in the arrangement of the spindle poles and proper separation of the chromosomes in a complex composed of Dynein-Dynactin-NuMA (DDN) [10]. Dynein is an energy supplier for eukaryotic cellular movements, and Dynactin serves as its cofactor. Studies have demonstrated NuMA-dynein role in DNA double-stranded break repair by homologous recombination related to BRCA proteins. NuMA is also involved in different cellular pathways including PLK1 activated cell division and caspase-mediated apoptotic response [11].

Our study aimed to investigate the association between SPAG5 and NuMA mRNA levels in breast cancer tumors. Since SPAG5 gene amplification was also involved in the pathogenesis of the breast cancer, we also intended to identify any association between the amplified genome and SPAG5 / NuMA mRNA levels.

## Methods

### Patients

Patients were selected between January 2017 and October 2018 by random sampling from Farmaniyeh Hospital and Sina Hospital, affiliated to Tehran University of Medical Sciences. Inclusion criteria were as follows: a new case of biopsy-confirmed breast invasive ductal carcinoma (IDC) with or without ductal carcinoma in situ (DCIS). Exclusion criteria were receiving any chemotherapy, radiotherapy, and hormone therapy. The patients signed a consent form approved by the ethics committee of Tarbiat Modares University with the registration number of IR.TMU.REC.1396.681. Eighty tissue samples including forty tumors and forty normal tissues adjacent to the tumor (NAT) were collected under the supervision of the breast surgeon. Patient’s clinicopathological characteristics comprising age, family history of cancer, lymph node involvement, tumor size, tumor grade, estrogen receptor (ER), progesterone receptor (PR), and human epidermal growth factor receptor 2 (HER2) statuses were recorded. Clinical stage was determined by TNM system including tumor size, lymph node involvement, and metastasis. Lymph node involvement was confirmed histologically. According to AJCC guideline, the molecular stage was defined using TNM, tumor grade, and ER, PR, and HER2 status. We further classified patients to breast cancer subtypes: luminal A, luminal B, HER2 positive, and basal-like. Ki67 index as a marker for tumor proliferation was applied to subtype luminal tumors by the cut-off value of 14%.

Since NATs were taken from the non-tumorous region of the same patient’s breast, for a more accurate comparison, we also included three samples of healthy breast tissues from individuals with no history of breast cancer. The reason for surgery in these healthy individuals was cosmetic restorations and they did not have any history of cancer in 1st and 2nd-degree family relatives.

### MCF7 and MDA-MB-231 cell line culture

Breast cancer cell lines were used as the control of real-time PCR and quantitative multiplex fluorescent PCR (QMF PCR). MCF-7 (IBRC C10082) and MDA-MB-231(IBRC C10684) were obtained from the National Cell Bank of Iran. Both cell lines were invasive breast ductal carcinoma bearing an epithelial-like morphology. Cell lines were cultured in the medium containing Dulbecco’s modified eagle medium (DMEM) and 10% fetal bovine serum (FBS).

### DNA and RNA extraction

After homogenization of 20 mg breast tissue, DNA and RNA were simultaneously extracted by Allspin^TM^ mini kit (GeneAll, Pishgam Biotech, Iran). DNA and RNA quantities were determined by spectrophotometry at 230, 260, and 280 nm. The observation of 18s rRNA and 28s rRNA bands in gel electrophoresis was in favor of appropriate RNA quality. cDNA synthesis was performed by 2X RT Pre-mix kit (BioFact, Noavaran Teb, Iran). M-MLV Reverse transcriptase, oligodT and random hexamer were simultaneously applied to increase cDNA synthesis efficacy.

### Real-time PCR

The expression of SPAG5 and NuMA genes were quantified by real-time RT PCR. To normalize the results, PUM1 was selected as the reference gene. PUM1 is a housekeeping gene with high and constant expression in normal and tumor breast tissue. Primer design for SPAG5, NuMA, and PUM1 genes was conducted by Primer3 and Oligo analyzer software and checked on BLAST (basic local alignment search tool) websites. To prevent genomic DNA amplification, all real-time PCR primers spanned exon-exon junctions. The detailed characteristics of real-time primers are provided in Table 1. Real-time PCR was performed by Applied Biosystem Step One system using SYBR Green dye. PCR efficiency was calculated by LinReg PCR software through linear regression analysis.

**Table 1:** Specific primers designed for quantitative real-time PCR

Gene	Sequence (5′_3′)	Length	Tm	GC%	Product (bp)
PUM1	F-CCTACCAACTCATGGTGGATGT	22	59.76	50	83
	R-AGCCAGCTTCTGTTCAAGACT	21	59.58	47	
SPAG5	F-AAGGAGAAGACTGAACAAGAGACC	24	59.96	45.8	119
	R-TCATCTGCCACTGCTGTCAAG	21	60.61	52.4	
NUMA	F-AGAGAGCAAACTAAGCAGGTGG	22	60.29	50	97
	R-CCTGGACAGCCTTCAGCTTCT	21	62.06	57.1	

*PCR* polymerase chain reaction, *Tm* melting temperature, *GC* guanine cytosine, *bp* base pair, *PUM1* pumilio RNA-binding family member 1, *F* forward, *R* reverse, *SPAG5* sperm-associated antigen, *NUMA* nuclear mitotic apparatus protein

### QMF PCR

SPAG5 gene amplification was examined through QMF PCR and biorientation of chromosomes in cell division 1 like 1(BOD1L), ATP/GTP-binding protein like 2 (AGBL2), and cytochrome P450 oxidoreductase (POR) were selected as internal control genes. The primers were designed for three parts of SPAG5 gene; 5′ end, middle and 3′ end of the gene. All internal controls and three SPAG5 primers comprised similar Tm (Table 2). To fluorescently label PCR fragments, universal primer was opted for as an efficient and cost-effective method. Universal primer sequence published by Roche company which does not have any specific complementary site in the human genome was selected [12]. Each fragment was amplified by 3 primers: tailed locus-specific forward primer, locus-specific reverse primer, and 6-FAM-labeled universal primer in a concentration ratio of 1:2:1. Due to the lower starting concentration of the tailed locus-specific primer, as PCR progress, it was gradually depleted and the 6-FAM labeled universal primer was subsequently included. AmpliTaq Gold^TM^ DNA polymerase (Ampliqone, Pishgam Biotech, Iran) with hot start activity was applied for a multiplex PCR reaction. Capillary electrophoresis was performed via 0.5 μl of the PCR products, 9 μl of formamide, and 0.5 μl of GeneScan-500 LIZ size standard (Applied Biosystems, Courtaboeuf, France). The fluorescent PCR products were heat-denatured, chilled on ice, and separated on an 8-capillary sequencer (3500 Genetic Analyzer, Applied Biosystems, France). The results were processed with GeneMarker 2.2 software, the peak area values were imported into an Excel (Microsoft) file, and the copy number of each fragment was calculated by the dosage quotient (DQ) method.

**Table 2:** Specific primers designed for QMF PCR

Gene	Ch.	Sequence	Length	Tm	GC%	Product (bp)
BOD1L	4	F-AATGCCTCCGCTTTCAGGC	19	61.05	57	151
		R-ATCACTTGGCAACTCACACATGG	23	61.62	47	
AGBL2	11	F-GCGAGCTGCATTCCATGCG	19	62.8	63	197
		R-TCCCAGCTTTGGAAACGCAC	20	61.45	55	
POR	7	F-AGCCACTTTGTGCCAGATCA	20	59.89	50	258
		R-TCCAGCACGTGTTCACATCA	20	59.89	50	
SPAG5-5′	17	F-CCCTAAGAAGCCCAAAATGCG	21	59.57	52	119
		R-TCCTGGAAAGTTGGGTCGAG	20	59.31	55	
SPAG5-Middle		F-AGGCTGCTCATCTGATTCATGC	22	61.07	50	394
		R-CAAGCTACCATCTGCCCACG	20	61.09	60	
SPAG5-3′		F-GTTAGGAAAGGGTCGAAAGGGC	22	61.2	54	451
		R-ACACCCTATCAAAAGTCTGTTCC	23	58.35	43	

*QMF PCR* quantitative multiplex fluorescent polymerase chain reaction, *Ch* chromosome, *Tm* melting temperature, *GC* guanine cytosine, *bp* base pair, *BOD1L* biorientation of chromosomes in cell division 1 like 1, *AGBL2* ATP/GTP-binding protein like 2, *POR* cytochrome P450 oxidoreductase, *SPAG5* sperm-associated antigen

### Statistical analysis

The sample size was calculated by Epi Info™ software. SPSS Statistic version 21 and GraphPad Prism version 8 were used for real-time data analysis. To determine the type of appropriate statistical method, data were analyzed by Kolmogrovo-Smirnov for distribution normality. Since the distribution of data was normal, the parametric test was used. Paired-sample *t* test was employed to compare SPAG5 and NuMA gene expression level between tumors and NATs. The links between clinicopathological status and mRNA levels were calculated via independent sample *t* test and one-way ANOVA. For QMF PCR result analysis, SPSS Statistic version 21 was used to determine the frequency of samples with gene amplification.

## Results

### Patient’s characteristics

The mean age of the patients was 55 ± 2 years. The minimum and maximum ages of the patients were 31 and 82 years. Thirteen patients (32%) had a family history of cancer, mainly breast or colon cancer. Detailed clinicopathological features of the patients are given in Table 3.

**Table 3:** Statistical analysis of clinicopathological variables related to SPAG5 and NuMA mRNA expressions

			*N* (%)	*t*	SSig. (2-tailed)	Std. Error	95% CI	
							Lower	Upper
SPAG5	Age	< 50 years	16(40)	− 1.05	0.29	1.19	− 3.67	1.15
		> 50 years	24(60)					
	Grade	I and II	30(75)	− 0.97	0.33	1.31	− 3.94	1.37
		III	10(25)					
	LN	Positive	18(45.5)	0.77	0.44	1.18	− 1.47	3.31
		Negative	22(55.5					
	FH	Positive	13(32.5)	0.78	0.43	1.25	− 1.55	3.53
		Negative	27(67.5)					
	ER	Positive	35(87.5)	− 0.19	0.84	1.79	− 3.98	3.27
		Negative	5(12.5)					
	PR	Positive	33(82.5)	− 0.02	0.98	1.56	− 3.20	3.12
		Negative	7(17.5)					
	HER2	Positive	7(17.5)	− 0.02	0.98	1.56	− 3.20	3.12
		Negative	33(82.5)					
NUMA	Age	< 50 years	16(40)	0.89	0.37	0.79	− 0.90	2.32
		> 50 years	24(60)					
	Grade	I and II	30(75)	− 1.91	0.06	0.84	− 3.32	0.09
		III	10(25)					
	LN	Positive	18(45.5)	− 1.0	0.32	0.78	− 2.37	0.79
		Negative	22(55.5					
	FH	Positive	13(32.5)	− 1.93	0.06	0.80	− 3.18	0.06
		Negative	27(67.5)					
	ER	Positive	35(87.5)	− 2.90	**0.006**	1.07	− 5.32	− 0.95
		Negative	5(12.5)					
	PR	Positive	33(82.5)	− 0.79	0.43	1.03	− 2.90	1.26
		Negative	7(17.5)					
	HER2	Positive	7(17.5)	− 0.79	0.43	1.03	− 2.90	1.26
		Negative	33(82.5)					

Independent sample *t* test was applied to compare the SPAG5 and NuMA gene expression levels according to clinicopathological features. Normal distribution of variables and equality of variances were checked by Kolmogorov-Smirnov and Levene’s test, respectively. The significance level was selected at 0.05.

*N* number, *t t* test, *sig* significance (*p* value), *Std* standard, *CI* confidence intervals, *SPAG5* sperm-associated antigen, *NuMA* nuclear mitotic apparatus protein, *LN* lymph node involvement, *FH* family history, *ER* estrogen receptor, *PR* progesterone receptor, *HER2* human epidermal growth factor receptor 2

Clinical stage was categorized by TNM system and the results revealed that stage I, stage II, and stages III/IV assigned to 30%, 47.5%, and 22.5%, of the samples, respectively. As indicated in Table 4, 75% of the samples were categorized in the molecular stage I, 12.5% in stage II, and 12.5% in stages III/IV. Further classification of breast cancer subtypes showed that luminal, HER2 positive, and basal-like comprised 85%, 5%, and 10% of tumors, respectively. Using Ki67 index analysis, we observed that luminal A allocated to 30% and luminal B to 55% of the tumors.

**Table 4:** Analysis of staging related to SPAG5 and NuMA mRNA expression. Clinical stage was determined by the TNM system (tumor size, lymph node involvement, metastasis). According to AJCC guideline, the molecular stage was defined using TNM, tumor grade and ER, PR and HER2 status

			*N* (%)	Sum of squares	df	Mean Square	*F*	*p* value
SPAG5	Clinical stage	Stage I	12(30)	102.71	2	51.35	4.50	**.018**
		Stage II	19(47.5)					
		Stages III and IV	9(22.5)					
	Molecular stage	Stage I	30(75)	2.74	2	1.37	.09	.90
		Stage II	5(12.5)					
		Stages III and IV	5(12.5)					
	Molecular subtypes	Luminal A	12(30)	34.11	3	11.37	.83	.48
		Luminal B	22(55)					
		HER2 positive	2(5)					
		Basal-like	4(10)					
NuMA	Clinical stage	Stage I	12(30)	4.03	2	2.02	.32	.72
		Stage II	19(47.5)					
		Stages III and IV	9(22.5)					
	Molecular stage	Stage I	30(75)	8.73	2	4.36	.72	.49
		Stage II	5(12.5)					
		Stages III and IV	5(12.5)					
	Molecular subtypes	Luminal A	12(30)	44.97	3	14.99	2.88	**.049**
		Luminal B	22(55)					
		HER2 positive	2(5)					
		Basal-like	4(10)					

One-way ANOVA was employed to analyze the difference between SPAG5 and NuMA gene expression levels related to clinical stage, molecular stage and molecular subtypes. Normal variable distribution and equality of variances were checked by Kolmogorov-Smirnov and Levene’s test, respectively. Our chosen significance level was 0.05

*N* number, *df* degree of freedom, *SPAG5* sperm-associated antigen, *NuMA* nuclear mitotic apparatus protein

### SPAG5 gene expression levels

Melting curve analysis of the PCR products showed a single peak indicating specific amplification of the desired gene fragment. To normalize the results, the Ct difference (ΔCt) of SPAG5 and PUM1 was computed. To compare SPAG5 gene expression in tumor with NATs, ΔΔCt was determined. The fold change was calculated by 2^−ΔΔCt^ method. The SPAG5 and PUM1 gene PCR efficiency was 1.99 obtained by LinRegPCR software.

**Fig. 1:**
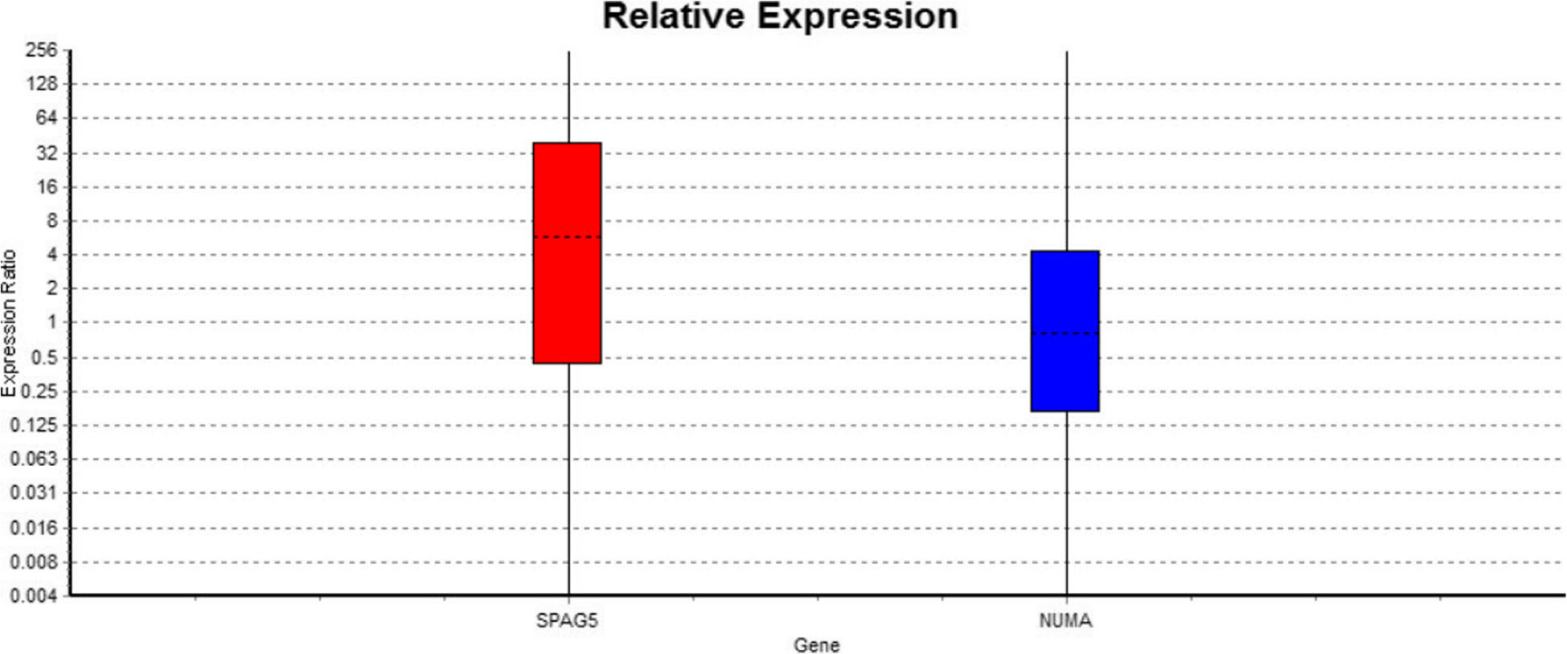
Fold changes of SPAG5 and NuMA genes calculated by the Relative Expression Software Tool (REST©). SPAG5 mRNA expression was increased 4.29 times in breast tumors than the normal adjacent tissues (*p* = 0.005). The mean fold change of NuMA gene expression in tumor relative to normal adjacent tissue was 0.821

The statistical analysis revealed a significant difference between SPAG5 gene expression levels in tumor relative to NATs (*p* = 0.005). SPAG5 gene expression was upregulated in tumor tissues with a mean fold change of 4.29 and the mean difference of 2.103 (Fig. 1).

Using independent sample *t* test, the SPAG5 mRNA levels were analyzed in correlation to age, tumor grade, ER, PR, and HER2 status. As indicated in Table 3, the results showed no statistically significant association between SPAG5 gene expression and these clinicopathological features.

However, correlation studies revealed a significant link between SPAG5 gene expression and clinical stage (*p* = 0.018). Since the clinical stage was defined by tumor size, lymph node involvement, and metastasis, we analyzed these parameters, separately. The patients’ tumors were categorized according to the size (0–2 cm, 2–4 cm, 4–7 cm), and SPAG5 gene expression was evaluated in the patients’ groups by one-way ANOVA analysis. The results demonstrated a significant link between SPAG5 mRNA levels and tumor size (*p* = 0.018). SPAG5 gene expression level was higher in larger tumors. Lymph node involvement and metastasis revealed no significant correlation to SPAG5 mRNA levels. The statistical analysis regarding molecular stage and molecular subtypes failed to show any significant association (Table 4).

### NuMA gene expression levels

The mean of Ct values for NuMA and PUM1 were analyzed for duplicate samples and modified based on reaction efficiency. The results of LinReg analysis indicated PCR efficiency of 1.99 for NuMA gene. The specificity of the PCR amplification was confirmed by melting curve graphs of each reaction. The mean fold change of NuMA gene expression in tumors relative to NATs was 0.821, which was not statistically significant (p=0.56). Further data analysis disclosed a significant association between NuMA gene expression and ER status (p=0.006). ER-negative tumors showed eight times less NuMA gene expression than ER-positive tumors. ER is one of the main parameters of molecular subtype classification, so as expected, this significant difference was also observed regarding the link between NuMA expression and molecular subtypes (*p* = 0.049). However, NuMA expression did not show any significant difference in the molecular stage or the clinical stage (Table 4).

The correlation study to other clinicopathological status revealed no significant link with age, tumor grade, lymph node involvement, and tumor size. No significant connection was observed regarding PR or HER2 status (Table 3).

### The correlation between SPAG5 and NuMA gene expression in tumor tissue

**Fig. 2:**
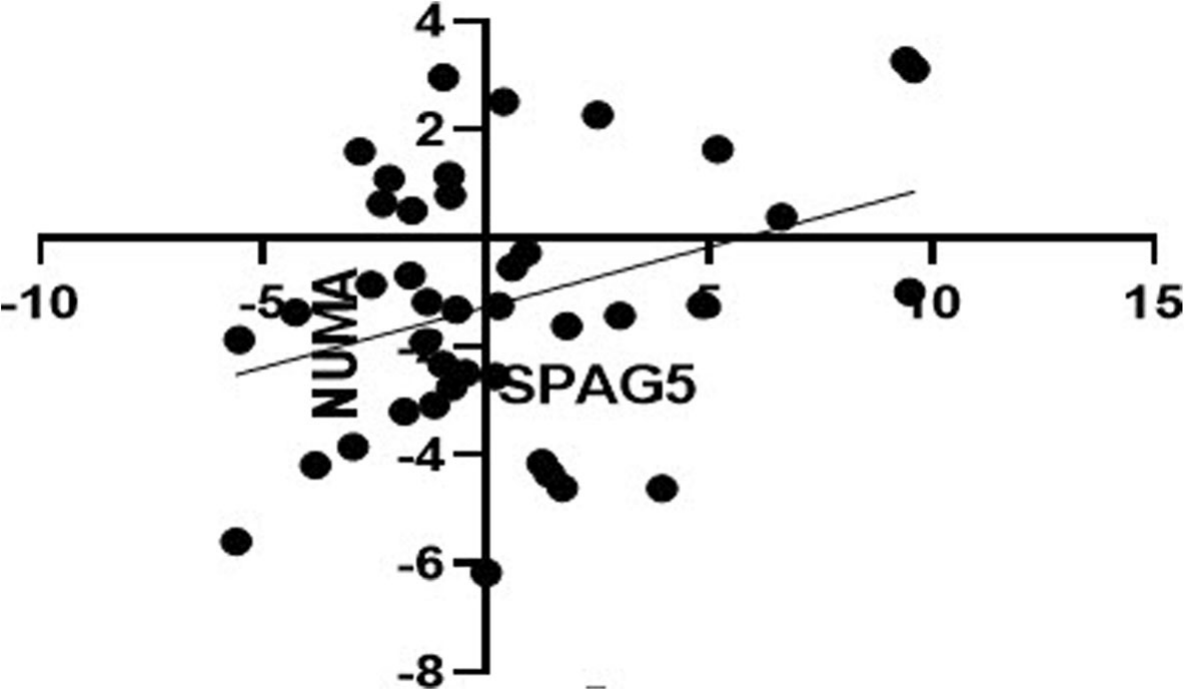
Pearson correlation of SPAG5 and NuMA expression levels in tumor samples. The *X* and *Y*-axes show ΔCt of SPAG5 and NuMA genes, respectively

The study of the correlation was conducted between SPAG5 and NuMA genes expression using the Pearson test. In Fig. 2, the *X* and *Y*-axes show ΔCt of SPAG5 and NUMA genes, respectively. The test results showed that there was no statistically significant correlation between the two mRNA levels (*r* = 0.33).

### QMF PCR results

**Fig. 3:**
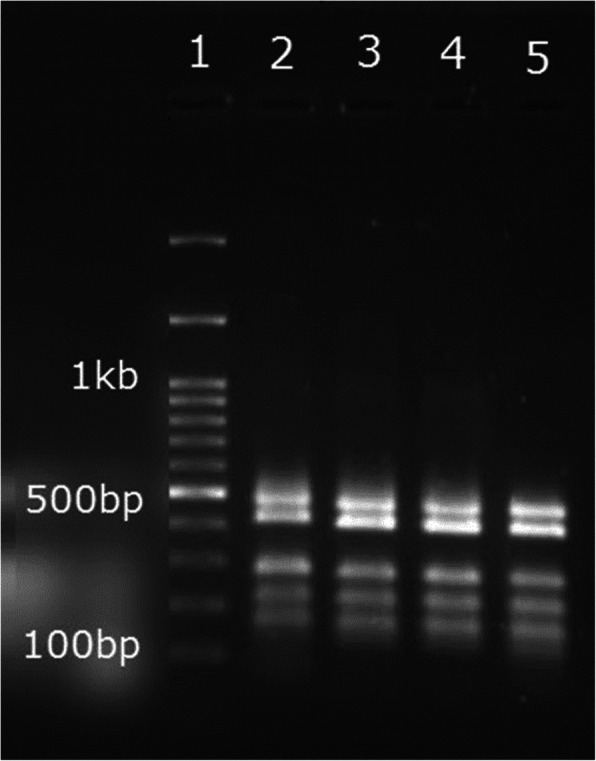
Gel electrophoresis of multiplex PCR. First well is loaded by ladder 100 bp and wells 2–5 are loaded with DNA of tumor tissues. Bands arrangement from biggest to smallest are 3′ end of SPAG5 (466 bp), middle of SPAG5 (409 bp), POR (273 bp), AGBL2 (212 bp), BOD1L (166 bp), and 5′ end of SPAG5 (134 bp)

**Fig. 4:**
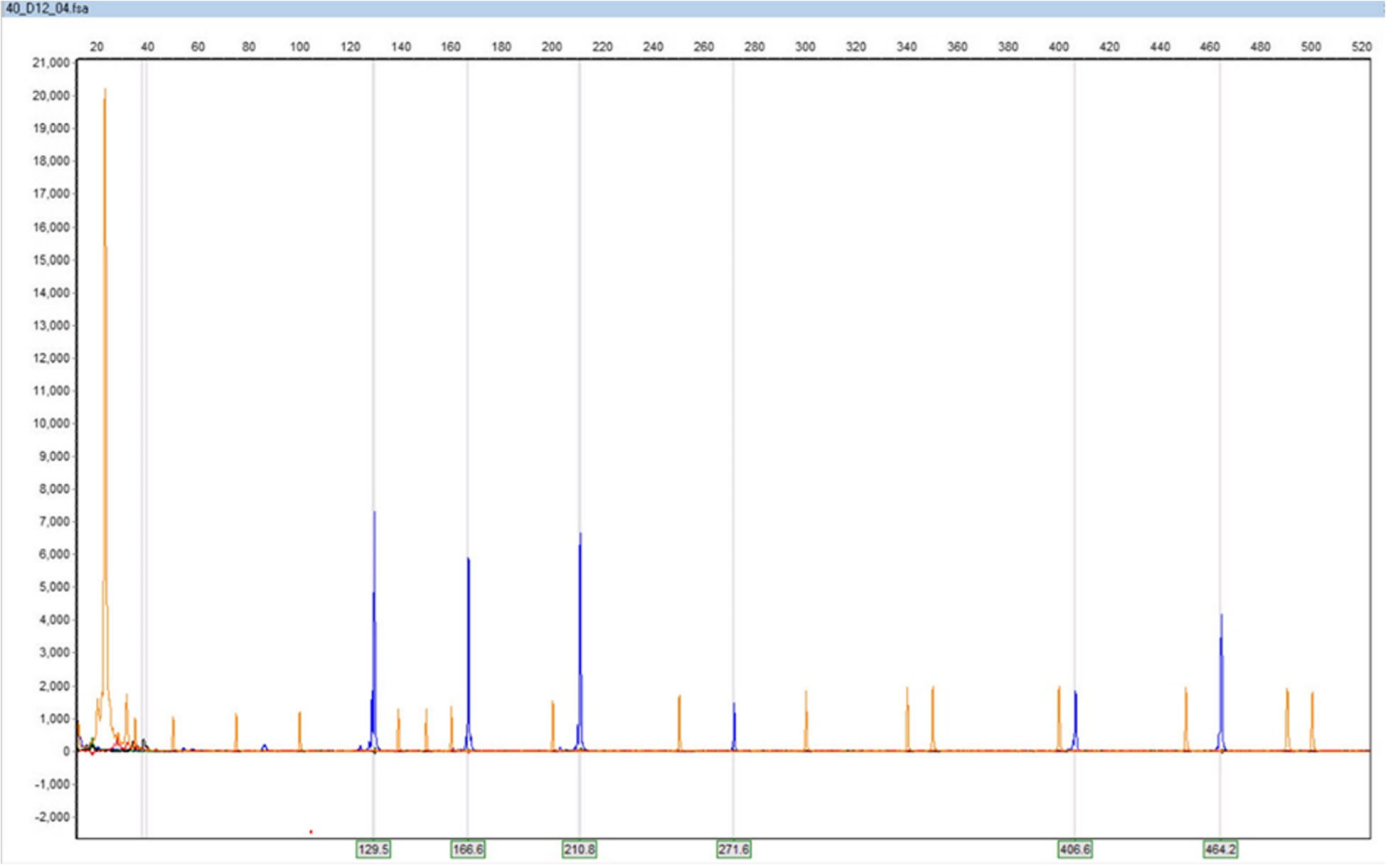
DNA fragments electropherograms in GeneMarker® software. The *X* and *Y*-axes represent DNA fragments length in bp and signals intensity as a relative fluorescent unit (RFU), respectively. The orange peaks were generated by standard size fragments and the blue peaks by SPAG5 DNA fragments and control genes. The order of the peaks from the largest to the smallest corresponds to 5′SPAG5, BOD1L, AGBL2, POR, middle SPAG5, and 3′SPAG5

The fragment analysis was performed following verified multiplex PCR. PCR products should have at least 20 bp length differences to be distinguishable in capillary electrophoresis (Fig. 3). We applied universal primer to avoid non-specific PCR products and amplify multiple loci in one PCR reaction. The copy number of each SPAG5 fragment was determined by calculating the DQ obtained by dividing the fragment peak area by the mean peak area of control genes. As demonstrated in Fig. 4, the *X* and *Y*-axes represents DNA fragments length in base pair (bp) and signal intensity as a relative fluorescent unit (RFU). The + 3SD and − 3SD values were considered as cut-off points. Values greater than 1.2 was considered amplified and values less than 0.8 as deletion according to previous publications [13].

MCF-7 and MDA-MB-231 cell lines showed complete SPAG5 gene amplification. While three normal breast tissues and two healthy donor blood samples which used as normal controls did not show any gene amplification. We calculated mean DQ of 1.28 and 1.33 for MCF-7 and MDA-MB-231, respectively. Six samples (17%) showed complete gene amplification in all three SPAG5 gene fragments with mean DQ of 1.54 comparing 10 samples (28%) which had no amplification in any fragment of SPAG5 gene (mean DQ = 0.96). The rest of the samples showed amplification in one or two parts of the gene with a mean DQ of 1.15.

## Discussion

The main purpose of this study was to investigate SPAG5 gene expression and amplification related to NuMA mRNA levels in breast cancer tissues. As the normal control sample, we used the same patient’s non-tumor breast tissue. It can be assumed that this seemingly healthy tissue, which was adjacent to a tumor, may have altered gene expression or amplification. To prevent this drawback, we attempted to sample at the farthest possible distance from the tumor as recommended by the cancer genome atlas (TCGA) [14]. TCGA mentioned that NAT sampling must be performed > 2 cm from the tumor margin. It has been reported that genetic changes could be detected in the normal margin of the tumor, but are rarely evident at distances greater than 1 cm [15]. In our study, we further evaluated the NATs, histopathologically, and we observed no morphological or molecular alterations. To evaluate possible changes in SPAG5 and NuMA gene expression in the NATs, three healthy breast tissues from women referred for cosmetic surgery were included in the study. The ΔCt of SPAG5 and NuMA genes in these three samples were the same as the mean of ΔCt of SPAG5 and NuMA genes in NATs indicating that the NATs did not bear noticeable expression changes in the studied genes.

Our results showed that SPAG5 mRNA expression was increased 4.29 times in breast tumors than the NATs. Correlation studies revealed a significant link between SPAG5 gene expression and tumor size with higher levels in larger tumors. Our results also showed a significant correlation between SPAG5 gene expression and clinical stage.

SPAG5 knockdown has shown cell growth suppression and apoptosis enhancement, while the increased SPAG5 expression promotes cell proliferation and inhibits apoptosis [4]. As previously mentioned, SPAG5 prevents apoptosis through PI3K/AKT/mTOR pathway and is similarly involved in Wnt/β-catenin signaling pathway activation via stimulation of Wnt and β-catenin expression [16]. Clinical studies have confirmed the role of SPAG5 in breast cancer prognosis and survival. It has been shown that patients with higher SPAG5 gene expression were at high risk of cancer [17]. SPAG5 was also proposed as a proliferative marker in early-stage of cancer [18]. Li et al. reported SPAG5 as an oncogene in basal-like (triple-negative) breast cancer by direct interaction with MYC-binding protein (MYCBP) which results in an increased c-MYC transcriptional activity. C-MYC regulates the expression of several genes involved in the process of cell growth and DNA repair, exhibiting high expression in basal-like tumors. Based on the obtained results, they considered SPAG5/MYCBP/c-MYC axis as a potential therapeutic target in the triple-negative patients [19]. However, our study did not reach similar results, and we observed no significant difference in SPAG5 gene expression in basal-like tumors.

The previous study had suggested a link between SPAG5 and NuMA during cell division [9]. We also analyzed the correlation between SPAG5 and NuMA gene expression in pair-wise comparisons. Our results yielded no significant correlation between SPAG5 and NuMA indicating independent expression of these two genes in the process of breast tumorigenesis. Although no link was found between the expression levels of these two genes, further studies could investigate the association of their proteins.

We investigated NuMA transcript levels expression in breast cancer tissues relative to NATs, and we observed decreased NuMA expression; however, it was not statistically significant. An interesting finding of our study was that NuMA expression in ER-positive was higher than ER-negative tumor specimens. It can be justified that NuMA expression increases in ER-positive cells since the ER requires nuclear matrix components such as NuMA for its transcriptional activity. Previous studies have suggested the association between ER intra-nuclear mobility and the dynamics of the nuclear matrix [20]. The main function of the ER is localized in the nucleus where it acts as a ligand-dependent transcription factor. It interacts with multiple intra-nuclear components including nuclear matrix proteins to regulate transcription of the estrogen-responsive genes. In the presence of a ligand, ER distribution shifts from the diffused to the discrete state, indicating the binding of the receptor to the nuclear matrix elements. Since NuMA is a part of the nuclear matrix components, it could be concluded that it plays a pivotal role in the mobility of ER [21].

Our results showed a significant association between NuMA mRNA levels and molecular subtypes which are classified based on ER, PR, and HER2 expression. Recently, using machine learning algorithms, Kothari et al. attempted to find potential genes, discriminating basal-like from other subtypes. They reported TBC1D9 as a candidate gene that its expression was linked to improved patient’s survival. Further analysis revealed different roles for TBC1D9 in organelle biogenesis and localization as well as recruitment of NuMA to the mitotic centrosome [22]. It has been shown that BRCA deficiency causes cortical asymmetry of NuMA-dynein complex in dividing cells which provoke genomic instability and aneuploidy [23]. A new role for NuMA was revealed related to diffusion regulation of P53-binding protein 1 (53BP1) which plays a prominent role in DNA repair. Higher NuMA mRNA levels were significantly linked to increased breast cancer patients survival even when specifically analyzed in basal-like tumors [24].

Association study using SNPs in nearly 16,000 genes showed that a 300-kb region on chromosome 11q13 encompassing the NuMA gene influences breast cancer risk. They suggested A794G variant as a susceptibility allele for breast cancer [25]. However, additional investigations did not support the role of NuMA variants as breast cancer susceptibility alleles [26].

The other phenomenon of interest in our study was the SPAG5 gene amplification. Applying QMF PCR to evaluate gene copy number variation (CNV), we observed complete amplification in 17% of tumor samples. These results were in line with the results of the Abdel-Fatah et al. that reported 10–19% SPAG5 gene amplification in breast tumors [1]. Defects in DNA replication or telomere dysfunction promote gene amplification which can lead to tumor progression in any stage of breast cancer [27]. Gene amplification confers a selective advantage during early-stage of cancer by increasing oncogenes expression in amplified regions. It has been shown that gene amplification is associated with more aggressive tumors as well as resistance to chemotherapy which can predict disease prognosis [28].

Despite the significant results, our study also has particular limitations that should be addressed in the future. The association of SPAG5 and NuMA at the protein levels and the molecular mechanism of their interaction need to be clarified in vitro and in vivo. Furthermore, due to the limited sample size, we included IDC samples in the study without considering the presence or absence of DCIS. It would be interesting to study the gene expression differences between pure IDC and IDC/DCIS.

## Conclusion

Our study highlights the role of increased SPAG5 gene expression and gene amplification in breast cancer. The results showed that in larger-sized tumors, the SPAG5 mRNA was increased. This evidence suggests that SPAG5 may be further studied as a therapeutic target in the treatment of breast cancer. Therefore, complementary studies can be suggested to investigate the role of the proteins that interact with SPAG5. We did not observe any significant correlation between SPAG5 and NuMA mRNA levels. However, our results indicated an association between NuMA and tumor ER expression status. This finding, reported for the first time in our study, could be the starting point for further studies on ER and cell division apparatus in breast cancer.

## Data Availability

The datasets used and/or analyzed during the current study are available from the corresponding authors upon reasonable request.
